# Optimization of thermoelectric performance in Sm-substituted SrSi₂ via carrier transport and lattice engineering

**DOI:** 10.1080/14686996.2025.2551486

**Published:** 2025-08-27

**Authors:** Vikrant Trivedi, Naohito Tsujii, Takao Mori

**Affiliations:** a Research Center for Materials Nanoarchitectonics (MANA), National Institute for Materials Science (NIMS), Tsukuba, Japan; b Graduate School of Pure and Applied Sciences, University of Tsukuba, Tsukuba, Japan

**Keywords:** Thermoelectric material, silicide, seebeck coefficient, electrical resistivity, thermal transport

## Abstract

The pursuit of sustainable thermoelectric materials requires the development of cost-effective and efficient compounds derived from earth-abundant elements. Here, we investigate the effects of samarium (Sm) substitution on the thermoelectric performance of SrSi₂ with compositions Sr_1-*x*_Sm_*x*_Si_2_ (*x* = 0, 0.05, 0.1, 0.15, and 0.2). Substituting Sm for Sr in SrSi₂ enhances the power factor at low substitution levels, while further substitution leads to a decrease, due to increased carrier scattering and reduced Seebeck coefficient. Introducing Sm substitution enhances phonon scattering through point defects, reducing lattice thermal conductivity. A peak figure of merit (*ZT*) of ~0.23 at room temperature is achieved for Sr₀.₉₅Sm₀.₀₅Si₂, demonstrating a 35% improvement over undoped SrSi₂. The weighted mobility of ~285 cm^2^/V·s and the tailored thermal transport emphasize the role of Sm substitution in modulating both electronic and thermal properties. These findings establish Sr_1-*x*_Sm_*x*_Si_2_ as a promising candidate for next-generation thermoelectric devices.

## IMPACT STATEMENT

A high *p*-type thermoelectric power factor of 2.9 mW/K^2^m and *ZT*  = 0.23 was achieved around room temperature by incorporating the mixed-valent Sm^2+^/Sm^3+^ ion to the earth-abundant and environmentally friendly material SrSi_2_.

## GRAPHICAL ABSTRACT

**Figure:**
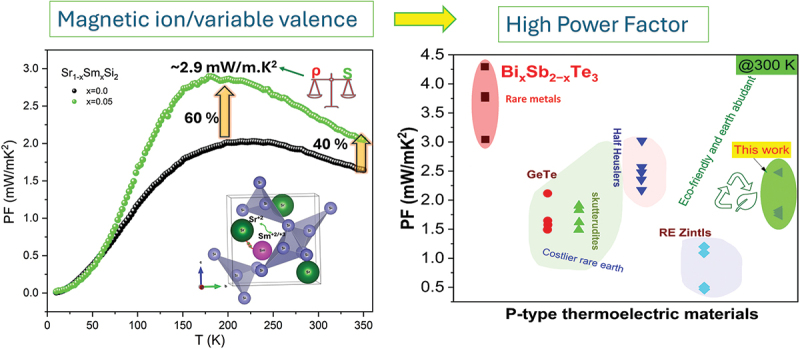

## 1. Introduction

The urgent need for developing renewable energy sources is more pressing than ever. Thermoelectric (*TE*) materials that can directly convert heat into electricity are crucial for energy harvesting and solid-state cooling technologies. This conversion relies on the movement of charge carriers and phonons. It offers several advantages, including minimal noise, no pollution, low maintenance requirements, and a long operational lifespan [1].

The effectiveness of a thermoelectric device in generating power or providing cooling is defined by the dimensionless figure of merit *ZT* (*ZT*=$S^{2}T/\kappa \rho$) of the materials. In this formula, *T* represents the absolute temperature, and *S*^*2*^*/ρ* is the power factor, where *S* is the Seebeck coefficient, *ρ* is the electrical resistivity, and *κ* is the thermal conductivity, in which the electron and phonon contributions are involved. Suitable thermoelectric materials must have significant Seebeck coefficient, high electrical conductivity, and low thermal conductivity. Practical thermoelectric materials have typical *ZT* values around 1. In recent decades, there has been renewed interest in finding new materials with excellent thermoelectric properties. These materials include rare-earth-filled skutterudites [2–4], MgAgSb [5], Mg_3_Sb_2_ [6–8], half-Heuslers [9]. Thus, the development of high-performance thermoelectric materials with earth-abundant and environmental-friendly elements is the current requirement.

Recently, alkaline-earth-metal disilicides have gained attention because they consist of non-toxic and naturally abundant elements. SrSi_2_ is recognized as a narrow-gap semiconductor, featuring a band gap of approximately 35 meV [10]. It may also be classified as a topological Weyl semimetal, as inferred by the band structure calculations [11,12]. Despite this understanding, literature on enhancing its thermoelectric properties is scarce. SrSi_2_ has two main phases: a cubic phase (*α*) that occurs at low temperatures and a tetragonal phase (*β*) that appears at high temperatures. The transition between these phases happens between 700 and 800 K. Theoretical calculations show that tetragonal *β*-SrSi_2_ has a metallic character, while *α*-SrSi_2_ has a band gap in the near-infrared range, making it useful for detecting infrared rays in silicon devices [13,14]. Achieving phase-pure *α*-SrSi₂ is typically challenging, and the presence of residual impurities can significantly influence the thermoelectric properties. For instance, α-SrSi₂ has been reported to exhibit a power factor (*S^2^/ρ*) ranging from approximately 1.6 to 2.4 mW/m·K^2^ at 300 K, depending on synthesis methods and microstructural modifications, with some values approaching those of commercial Bi₂Te₃-based thermoelectric materials [12,15,16]. The power factor (*PF*) of various doped SrSi₂ compositions exhibits notable improvements due to their high electrical conductivity and Seebeck coefficient. However, the material maintains a relatively high thermal conductivity of 5 to 6 W/m·K at room temperature [17]. The high thermal conductivity of pure *α*-SrSi_2_ results in a moderate dimensionless figure of merit (0.05–0.15) at room temperature. Nevertheless, *α*-SrSi_2_ is recognized for having the highest *ZT* among *p*-type silicides at room temperature. DFT calculations suggest an optimal *ZT* of approximately 0.77 at 300 K and nearly 1 at 50 K [18]. Hence, further optimization of thermoelectric properties is necessary. Several strategies were earlier applied in the case of *α*-SrSi_2_ by doping iso-electronic atoms (Ca, Ba, and Ge), electron and hole doping on Sr and Si sites (Y and Al), and processing techniques for nanostructuring (ball milling and melt spinning) [19]. Nanostructuring and doping are the most effective methods for optimizing phonon scattering in the lattice, reducing thermal conductivity without affecting the power factor.

In this study, we rigorously explore the impact of Sm substitution on the thermoelectric properties of SrSi_2_. Since Sm has a larger atomic size, its addition may significantly affect thermal conductivity due to mass/strain fluctuations. Also, replacing Sr with Sm could modify the band structure, as Sm has an extra electron in its valence shell compared to Sr. Furthermore, rare-earth elements such as Ce, Sm, Eu, and Yb may sometimes show valence instability because of dual-valence states (Ce^3+^/Ce^4+^, Sm^2+^/Sm^3+^, Eu^2+^/Eu^3+^, Yb^2+^/Yb^3+^). In that case, it potentially brings the Fermi level closer to a sharp peak in the density of states (DOS) [20–24]. Additionally, Sm, a magnetic ion, can generate magnetic entropy to enhance the Seebeck coefficient due to the degeneracy of its electronic structure [25]. We thus investigated the effect of Sm substitution in SrSi_2_, namely, Sr_1-*x*_Sm_*x*_Si_2_ (*x* = 0, 0.05, 0.1, 0.15, and 0.2) on the thermoelectric properties. This study provides a pathway for leveraging the dual-valence characteristics of rare-earth ions in SrSi₂, potentially enhancing its thermoelectric properties.

## 2. Experiments

### 2.1. Sample preparation

The samples of the polycrystalline Sr_1-*x*_Sm_*x*_Si_2 (_where *x* = 0, 0.05, 0.1, 0.15, and 0.2) were prepared using the Ar arc melting, ball milling, and spark plasma sintering route. For starting materials, Sr (3N, Furuuchi chemical, chunk), Sm (3N, Furuuchi chemical, chunk), and Si (10N, Furuuchi chemical, chunk) were used. The starting materials were placed in a water-cooled copper hearth under an argon atmosphere in the arc furnace. Multiple melting cycles were carried out to ensure homogeneity. To maintain the correct stoichiometry, an additional amount of Sr was added to compensate for its evaporation during the arc-melting process.

The arc-melted ingots were crushed in a WC-lined mortar pestle and ball-milled under an Ar atmosphere using a WC grinding medium by a planetary ball mill (Pulverisette-6, Fritsch, Germany) at a speed of 300 rpm for 3 hours. The milled powders were loaded in a graphite die under an Ar glove box and pelletized by the spark plasma sintering (SPS) with an SPS-1080 (Syntex Inc., Japan) at 1073 K and 80 MPa uniaxial pressure for 15 min in a static vacuum (2 × 10^−2^ Pa).

### 2.2. Sample characterization

Powder X-ray diffraction was measured using a RINT TTR-3 diffractometer (Rigaku Co., Akishima, Tokyo, Japan) with Cu *K*_α_ radiation. The diffraction data were refined using the Rietveld method through FullProf Suite, with Si (NIST 640d) employed as an external standard [26]. SEM and Electron backscattered diffraction (EBSD) measurements were performed using a FE-SEM (JSM-7001F, JEOL Ltd.). The EBSD measurements were done at 15 kV with a step size of 0.2 µm. The EBSD sample preparations were done by mechanical polishing to 0.1 μm with diamond paste and then ion milling. Chemical compositions were evaluated by using energy dispersive spectroscopy (EDS). X-ray photoelectron spectroscopy (XPS) measurements were conducted in a UHV system using a PHI 5000 VersaProbe II spectrometer (ULVAC-PHI, Japan). The thermoelectric properties *ρ*, *S*, and *κ* data were measured at temperatures ranging from 10 to 350 K using a Physical Property Measurement System (PPMS, Quantum Design) with the Thermal Transport Option (TTO) and the four-probe method. Bar-shaped samples with typical dimensions of 3 mm × 3 mm × 10 mm were used for these measurements. Corrections were made for heat loss due to radiation effects and dissipation through the leads to account for thermal conductivity data. The detailed procedure is documented elsewhere [27]. The room temperature Hall resistivity *ρ*_xy_(*H*) was measured under a magnetic field *H* ranging from −7 T to + 7 T at intervals of 1 T. The Hall coefficient (*R*_H_) was evaluated from the least-squares fitting of the antisymmetric part of *ρ*_xy_(*H*), [*ρ*_xy_(+*H*) - *ρ*_xy_(-*H*)]/2, against *H* to exclude the longitudinal contribution due to a possible misalignment of the Hall contacts. The carrier concentration and mobility were derived by *n*_H_ = 1/(*eR*_H_) and *μ*_H_ = *R*_H_/*ρ*, respectively. The measurement uncertainties were primarily based on the TTO system specifications. The typical accuracy for thermal conductivity (*κ*) is within ± 5% or the minimum detection limit, depending on the temperature range. The Seebeck coefficient (*S*) uncertainty is ± 5%, ±0.5 μV/K, or ±2 μV in voltage, whichever is greater. While the precision of resistivity (*ρ*) measurements is typically better than 0.01% for a resistance of 1 Ω at a measurement current of 200 μA, the overall uncertainty in *ρ* is predominantly governed by errors in measuring sample dimensions and contact area. These geometric uncertainties are estimated to result in an overall ± 5% error in resistivity. The uncertainty in the calculated thermoelectric figure of merit (*ZT*) is estimated to be ± 20%, assuming ± 5% errors in *κ*, *S*, and resistivity (*ρ*), with negligible contribution from temperature measurement errors. Error bars have been added to the relevant figures for clarity; these uncertainties were carefully considered during the analysis and interpretation of the thermoelectric performance.

## 3. Results and discussion

### 3.1. Phase formation and microstructure evolution

**Figure 1.**
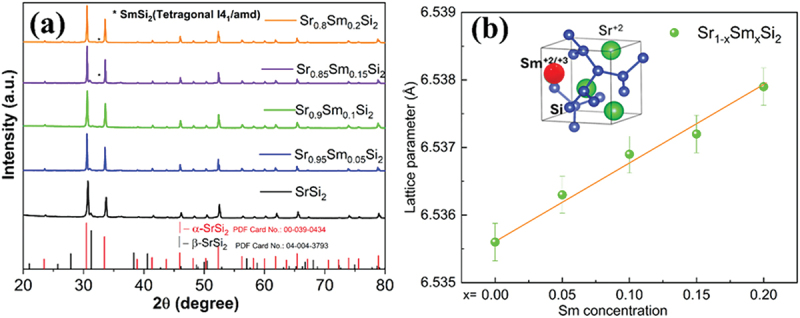
X-ray diffraction pattern of (a) SPSed Sr_1-*x*_Sm_*x*_Si_2_ samples with the peak corresponding to the *α*-SrSi_2_ and *β*-SrSi_2_ phases, impurity peaks at *x* = 0.15 and 0.2, marked by asterisks. (b) Lattice constant as a function of Sm content in Sr_1-*x*_Sm_*x*_Si_2_ samples. The inset in panel (b) illustrates the crystal structure of *α*-SrSi_2_.

**Figure 2.**
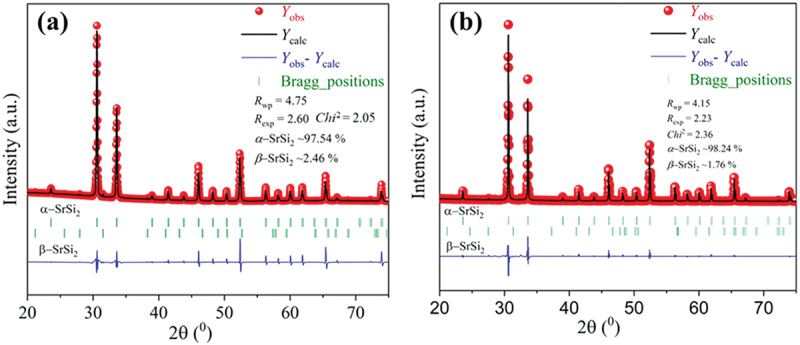
(a-b) XRD patterns of SrSi_2_ and Sr_0.95_Sm_0.05_Si_2_ SPSed samples with the Rietveld refined results and phase fractions.

Figure S1 (a, b) and Figure 1(a) show the powder X-ray diffraction (XRD) patterns of the Ar arc-melted, ball-milled, and SPSed samples in the range of 10° ≤ 2*θ* ≤80°, respectively. Each XRD result can be indexed to match the space group *P4*_*3*_*32* (No. 212). The XRD patterns of ball-milled powders in Figure S1(b) suggest that nanostructuring and disordering occurred due to ball milling, as evidenced by the broad XRD peaks. However, even after SPS, all the samples contained a minor portion of the tetragonal *β*-phase. The lattice parameter of the SPSed samples was estimated through Rietveld refinement of the XRD pattern (Figure 2(a,b)), which is plotted in Figure 1(b) as a function of the Sm concentration. The lattice parameter increases linearly with *x*, indicating that Sm atoms effectively replace Sr. The XRD data were further analyzed using a modified Williamson-Hall (W-H) method to estimate crystallite size, dislocation density, and lattice strain. Further details are available in the supporting information file. Figure S1(c) shows the average values of the samples’ dislocation density and crystallite size.

**Figure 3.**
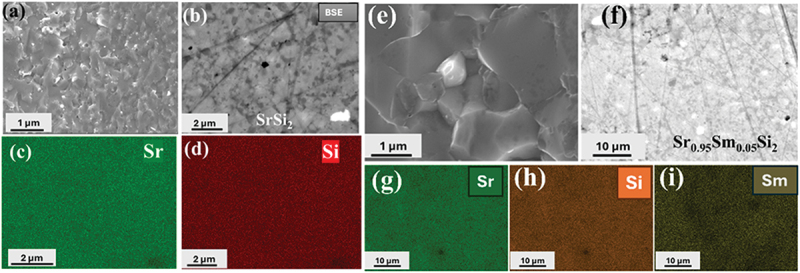
SEM image at the fractured surface of SrSi_2_ (a) and Sr_0.95_Sm_0.05_Si_2_ (e). BSE images at the polished surfaces of SPSed SrSi_2_ (b) and Sr_0.95_Sm_0.05_Si_2_ (f), and the respective EDS elemental mapping (c, d) for SrSi_2_ and (g-i) for Sr_0.95_Sm_0.05_Si_2_ (f).

We performed scanning electron microscopy with energy-dispersive X-ray spectroscopy (SEM-EDS) on the polished and fractured surfaces of Sr_1-*x*_Sm_*x*_Si_2_ to investigate the sample composition and morphology. Figures S2(a) and S3(a) present backscattered electron images (BEI) of arc-melted SrSi_2_ and Sr_0.85_Sm_0.15_Si_2_ samples, revealing three distinct regions: light, dark, and black. Elemental mapping of the arc-melted sample, as shown in Figures S2(b-c), indicates that the light areas correspond to the primary SrSi_2_ phase. In Figure S3(b-d), the white regions represent the Sm-rich impurity phase, while the gray and black regions are identified as the primary SrSi_2_ phase. Figures 3(a,e) display SEM images of fractured surfaces of two SPSed SrSi_2_ and Sr_0.95_Sm_0.05_Si_2_. The images reveal dense samples (~98% relative density) with no noticeable porosity. In Figures 3(b,f), the polished surfaces of the samples appear homogeneous, with no observable precipitated phases. Figures 3(c,d,g–i) show the EDS mapping of Sr, Sm, and Si for the SPSed SrSi_2_ and Sr_0.95_Sm_0.05_Si_2_ samples. These mappings confirm that all elements are uniformly distributed. Figure S4 shows BEI images of SPSed Sr_0.85_Sm_0.15_Si_2_ S4(a) and Sr_0.8_Sm_0.2_Si_2_ S4 (e). The lighter regions indicate a significant presence of Sm-rich secondary phases, as seen in the XRD analysis. EDS analysis results (Table 1) demonstrate that the primary elements in the samples match their nominal compositions.

**Table 1.** Energy-dispersive X-ray spectroscopy (EDS) results of Sr_1-*x*_Sm_*x*_Si_2_ SPSed samples, showing nominal vs actual composition of samples.

*x*_Nominal_	Sr (at. %)	Sm (at. %)	Si (at. %)	*x*_Actual_
0.00	32.7	–	67.3	0.00
0.05	30.6	2.1	67.2	0.07
0.10	29.6	3.1	67.3	0.09
0.15	29.1	4.0	66.9	0.12
0.20	26.9	6.2	66.9	0.19

**Figure 4.**
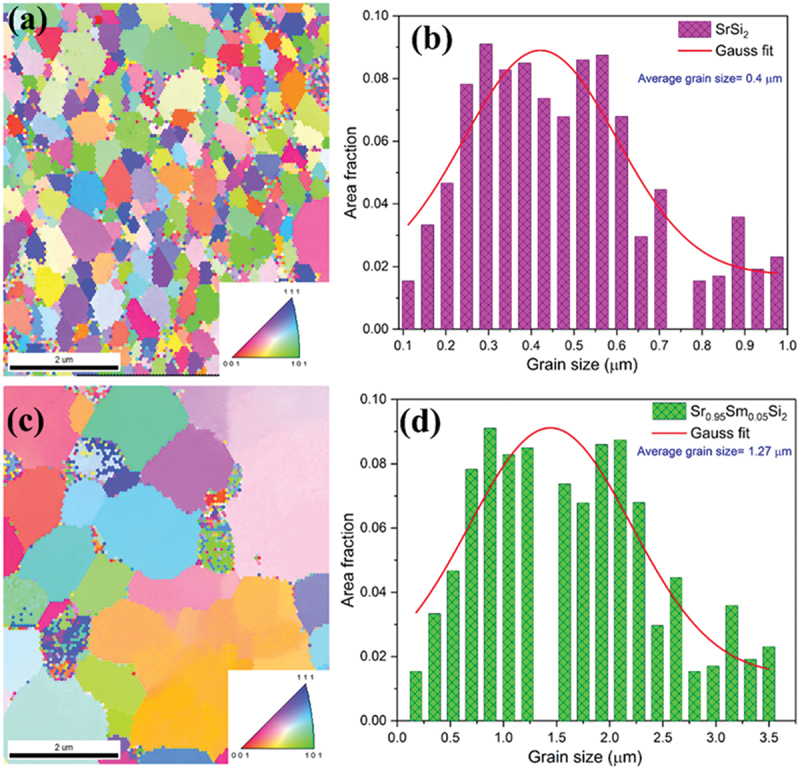
Electron backscatter diffraction (EBSD) patterns and grain size distribution for the undoped SrSi_2_ and Sr_0.95_Sm_0.05_Si_2_ samples. (a, c) and (b, d) are Euler images and histograms of the grain size of SrSi_2_ and Sr_0.95_Sm_0.05_Si_2_ samples, respectively. The insets in (a) and (c) are color-coded inverse Pole images.

EBSD analysis was performed on undoped SrSi₂ and Sm-substituted (*x* = 0.05) samples to study the effect of Sm substitution on local crystallography. Figure 4 shows orientation maps with colors representing Miller indices. Black spots signify unresolved areas caused by higher local strains or surface porosity that were excluded from the analysis. The Euler images (Figure 4(a,c)) reveal well-crystallized, randomly oriented grains. The grain size histograms (Figure 4(b,d)) indicate an increase in the average grain size from 400 ± 25 nm for SrSi₂ to 1.3 ± 0.4 μm for Sr₀.₉₅Sm₀.₀₅Si₂, which is likely a result of Sm substitution influencing grain boundary mobility and crystallization kinetics, as rare-earth doping has been reported to reduce grain boundary mobility through solute drag and lattice strain effects [28]. However, grain size variation and the inability of EBSD to distinguish sub-grains separated by low-angle boundaries must be considered [29].

**Figure 5.**
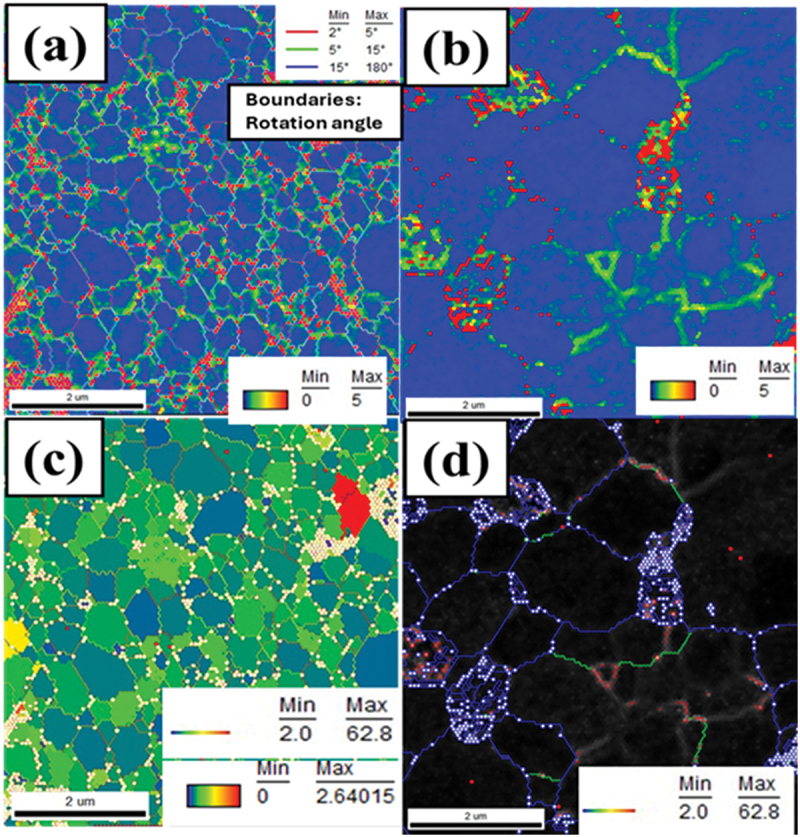
EBSD results of kernel average misorientation (KAM) maps of (a) SrSi_2_ and (b) Sr_0.95_Sm_0.05_Si_2_ sample, EBSD measured KAM plots of the sample showing higher dislocation density near the low angle boundaries. (c, d) grain boundary maps with grain orientation spread. In (c, d), color-coded grain boundaries with local misorientation angles are classified into several angular ranges.

Figure 5 illustrates local misorientation angles between adjacent grains and Kernel Average Misorientation (KAM) plots, providing insights into lattice distortions and dislocation density. High KAM values, particularly near grain boundaries, indicate significant dislocation density [30]. As discussed below, microstructural features like grain size, misorientation, and dislocation density critically impact thermoelectric performance by balancing enhanced phonon scattering with preserved electrical conductivity.

### 3.2. Thermoelectric properties

#### 3.2.1. Electrical resistivity

**Figure 6.**
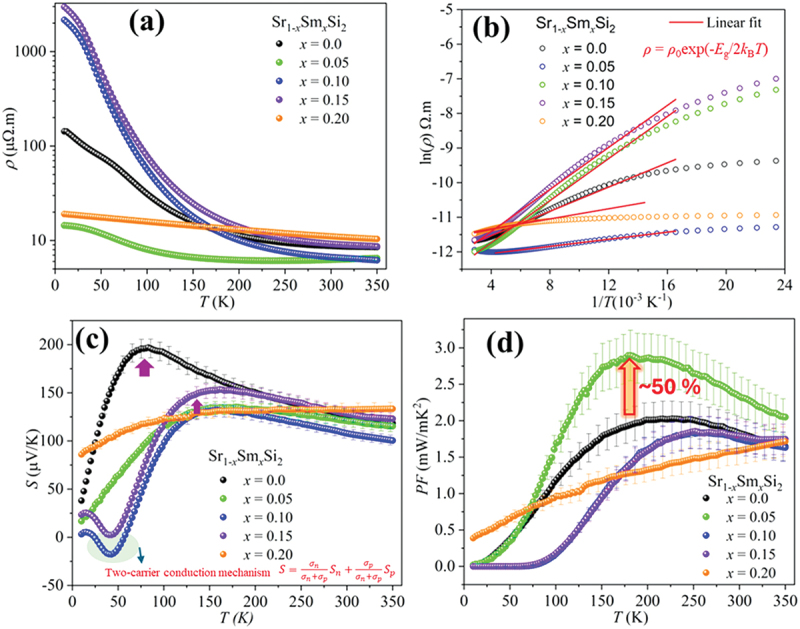
(a) Temperature dependence of electrical resistivity *ρ*(*T*) for Sr_1-*x*_Sm_*x*_Si_2_. (b) ln(*ρ*) vs 1/*T*. Solid red lines represent the fitting to the equation *ρ* = *ρ*_0_exp(-*E*_g_/2*k*_B_*T*), where *ρ*_0_ is a constant, *E*_g_ is the band gap, and *k*_B_ is the Boltzmann constant. (c) Seebeck coefficient (*S*) and (d) power factor (*PF*) of Sr_1-*x*_Sm_*x*_Si_2_ samples. The estimated relative error in *PF* is ± 15%, while the estimations of *S* and *ρ* have errors of ± 5%, as indicated by the representative error bar in figures.

The temperature-dependent electrical resistivity (*ρ*) for Sr_1-*x*_Sm_*x*_Si_2_ (0.0 ≤ *x* ≤0.2) is presented in Figure 6(a). The substitution of Sm for Sr reduces the electrical resistivity. The room temperature electrical resistivity is lower than reported in the previous literature [19,31,32]. All samples show a negative temperature coefficient of resistivity below 180 K. It has been observed that the room-temperature resistivity first decreases for Sm content up to *x* = 0.10 before increasing with further substitution. Room-temperature resistivity (*ρ*_RT_) of the Sr_0.95_Sm_0.05_Si_2_ was found to be decreased to (∼6.23 μΩ.m) from that of undoped SrSi_2_ (∼8.70 μΩ.m). In the case of Sr_0.8_Sm_0.2_Si_2,_ the absolute value of electrical resistivity increased, which suggested the effect of optimum electron doping and the role of secondary phases. The presence of secondary metallic phases such as *β*-SrSi₂ and SmSi₂ in the sample can lead to an increase in the overall electrical resistivity despite their intrinsic metallic nature [33,34]. This counterintuitive behavior arises primarily from enhanced electron scattering at the interfaces between the primary thermoelectric matrix and the metallic inclusions. These secondary phases typically form incoherent or semi-coherent boundaries with the host matrix, which act as scattering centers for charge carriers. The resulting carrier scattering, both elastic and inelastic, disrupts the charge transport pathways, thereby increasing resistivity. Additionally, compositional and structural inhomogeneities introduced by the impurity phases can lead to potential barriers or localized states, further hindering carrier mobility [35]. Thus, even though *β*-SrSi₂ and SmSi₂ are metallic, their dispersion within the matrix contributes to resistivity enhancement due to interfacial scattering effects and disruption of carrier percolation pathways. The increase in the absolute value of resistivity confirms a typical extrinsic behavior of metal/semi-metal. Imai et al. [10] also noted a decrease in electrical resistivity from 30 to 370 K, followed by a slight increase, which aligns closely with our results. Hashimoto et al. [32] and Aoyama et al. [36] also observed similar trends in samples made from commercial SrSi_2_ chunks. Similarly, as Singh et al. showed, replacing Sr with a larger atomic radius, which would induce a negative chemical pressure in the system, can alter the band characteristics [15]. The band gap of the samples at semiconducting temperature regions was calculated by fitting the *ρ*–*T* data employing the Arrhenius equation *ρ* = *ρ*_0_exp(-*E*_g_/2*k*_B_*T*), where *ρ*_0_ is a constant, *E*_g_ is the band gap, and *k*_B_ is the Boltzmann constant. The experimental data were analyzed by fitting into a linear equation involving ln(*ρ*) and 1/*T*, shown in Figure 6(b). *E*_g_ for undoped SrSi_2_ was obtained as 25 meV, consistent with the previous literature [11,15,32]. The value of *E*_g_ increases for lower Sm content (*x* = 0.05, 0.1) and decreases for higher Sm substitution levels (*x* = 0.15, 0.2) (see Table 2), suggesting the system became more metallic when doped heavily. Additionally, the presence of mixed-valence Sm^2 +^/Sm^3 +^ may not only influence carrier concentration but could also induce cooperative structural distortions or local symmetry changes within the lattice. While such changes may not be easily detected by conventional XRD due to their subtle or short-range nature, they could still impact the electronic structure and transport behavior [20,22]. To further investigate the possible valence states of Sm, we performed X-ray photoelectron spectroscopy (XPS) measurements for samples with *x* = 0.05 and *x* = 0.15. These measurements help clarify the presence of mixed-valence Sm^2 +^/Sm^3 +^ and their potential role in influencing both the electronic structure and local environment of the host lattice (Figures S5 and S6). The results agree with previous reports (see ESI) [37]. Due to a slight difference in the binding energies of Sm*^2 +^* and Sm*^3 +^* , it was only possible to qualitatively deconvolute the peaks. Therefore, within the scope of this work, determining the exact ratio of Sm^2 +^/Sm^3 +^ is not feasible.

**Table 2.** Band gap (*E*_g_), resistivity (*ρ*) at 10 K, 300 K, carrier concentration (*n*_H_), carrier mobility *(μ*_H_), and the temperature at which *S* has a maximum value.

*x*	*E*_g_ (meV)	*ρ*_10K_ (μΩ.m)	*ρ*_300K_ (μΩ.m)	*n*_H_ (10^20^ cm^−3^)	*μ*_H_(cm^2^V^−1^s^−1^)	*T*^S^_max_ (K)
0.00	25.8	142.2	8.7	0.96	73.6	85.5
0.05	31.5	14.4	6.2	1.04	99.6	181.2
0.10	24.4	2165	6.4	1.25	74.8	158.5
0.15	23.6	2988	9.1	0.33	201.9	163.6
0.20	23.1	19.05	11.02	0.18	308.0	163.6

Nevertheless, these resistivity trends align with Kuo et al. [31] and Shiojiri et al. [11], who state that the metallic behavior of highly doped samples at high temperatures may be due to impurities and defects. We also performed ball-milling to make fine powders, as that process is also known for generating defects like dislocations. Figure 5 shows that higher KAM values near grain boundaries and misoriented grains often indicate a greater defect density. Interestingly, the electrical resistivity is highly sensitive to defects known as scattering centers [38,39]. These observations indicate that the effects of variable valence state, along with secondary phases and tailored microstructure, play a crucial role in the band characteristics of Sm-substituted SrSi_2_, as indicated also by band structure calculations [11,14].

#### 3.2.2. Seebeck coefficient, power factor, and weighted mobility

The temperature dependence of the Seebeck coefficient (*S*) of Sr_1-*x*_Sm_*x*_Si_2_ SPSed samples is shown in Figure 6(c). The positive sign of *S* indicates that hole-type carriers are dominant, consistent with a small hole pocket near the Fermi level [40]. For each composition, the Seebeck coefficient shows a broad maximum below 150 K, a usual feature arising from thermally excited electrons moving across the gap or pseudo gap [41,42]. Doping induces a higher temperature shift in the broad maximum of *S* upon increasing Sm. The room-temperature value of *S* ~135 μV/K for SrSi_2_ is consistent with the previous reports [15,32,41]. The absolute *S* decreased with increasing temperature, probably due to the increase of carriers by thermal excitation [11,19]. It is also worth mentioning that substituted semiconductors obey a two-carrier conduction mechanism. Accordingly, the total *S* can be expressed as

(1) $$S=\frac{\sigma_{n}}{\sigma_{n}+\sigma_{p}}S_{n}+\frac{\sigma_{p}}{\sigma_{n}+\sigma_{p}}S_{p}$$

*S*_*n,p*_ and *σ*_*n,p*_ represent the Seebeck coefficients and electrical conductivities for the *n*- and *p*-type carriers. Subsequently, the signs of *S*_*n*_ and *S*_*p*_ are opposite, as governed by electron and hole transport. In the case of narrow-gap semiconductors or semimetals, thermal excitation of charge carriers can lead to the bipolar effect. For *x* = 0.10 and 0.15, the low-temperature Seebeck coefficient is negative, with peaks observed around 50 K, which can be qualitatively attributed to two-carrier conduction. As shown in Figure 6(c), the Sm-substituted samples exhibit dominant *n*-type carriers at low temperatures due to electron doping. At higher temperatures (above 100 K), the transport properties gradually become dominated by holes, most likely because the hole mobility is higher than that of electrons, as similarly reported in Y-substituted SrSi₂ systems by Lue et al. [17]. However, it is important to note that the samples with higher Sm content (*x* ≥0.10) contain significant metallic secondary phases such as *β*-SrSi₂ and SmSi₂, which may contribute to the observed electrical transport. Therefore, the explanation based solely on intrinsic doping and bipolar effects is not sufficient, and the complex multi-phase nature of these samples must be considered when interpreting the transport behavior.

**Figure 7.**
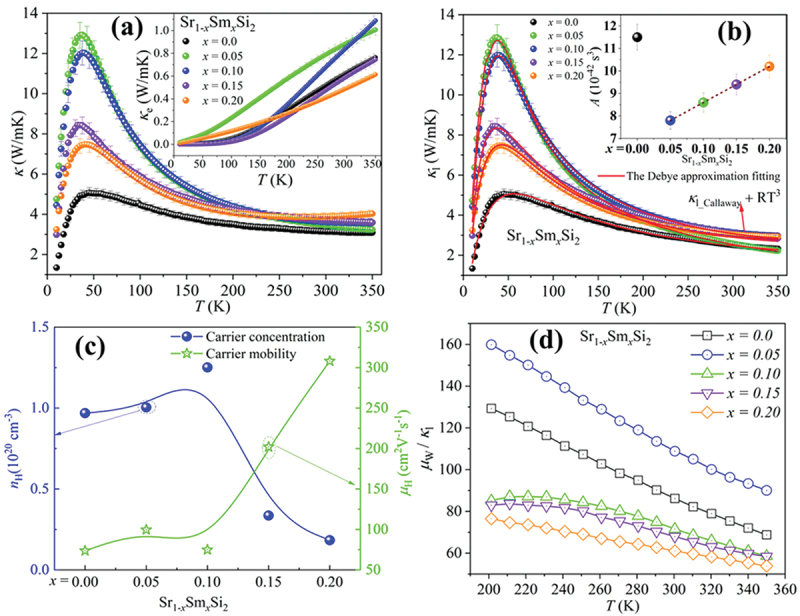
(a) Temperature dependence of the total thermal conductivity *κ*(*T*) for Sr_1-*x*_Sm_*x*_Si_2_. The inset in (a) shows *κ*_e,_ calculated by the Wiedemann – Franz law. (b) Lattice thermal conductivity (*κ*_l_) for Sr_1-*x*_Sm_*x*_Si_2_ as a function of temperature. Each solid curve represents the calculated *κ*_l_ using eqs. (4) and (5). The inset shows concentration-dependent point defect scattering parameter *A* of Sr_1-*x*_Sm_*x*_Si_2_ samples. (c) The carrier mobility and carrier concentration versus Sm content at 300 K, (d) the quality factor *μ*_W_/*κ*_l_ of Sr_1-*x*_Sm_*x*_Si_2_.The error bars in Figure (a-b) indicate an estimated uncertainty of ± 5% in the measurements of *κ*.

To gain deeper insight into the electrical transport properties, room-temperature carrier concentration and mobility were measured as a function of Sm content in Sr_1-*x*_Sm_*x*_Si_2,_ as shown in Figure 7(c) and Table 2. The samples demonstrate higher carrier concentrations than those previously reported in the literature [10,16]. This difference is likely due to variations in synthesis routes, Sr deficiency confirmed by EDS, and the presence of metallic secondary phases, all of which can influence the effective carrier density. Additionally, the grain size difference between the undoped (~400 nm) and Sm-substituted (~1.5 µm) samples suggests that grain boundary scattering may contribute more significantly to the Hall effect measurements in the undoped samples with smaller grains. In contrast, the larger grain sizes in substituted samples reduce the influence of grain boundaries, likely providing more reliable carrier concentration values. The carrier concentration follows a trend similar to *S*’ values, increasing up to *x* = 0.10 and then decreasing for higher *x*. We believe that at a certain level of Sm content, the density of thermally activated carriers becomes more significant than that of extrinsic carriers from impurities. To further understand the impact of Sm substitution, we applied the Pisarenko relation at 300 K, analyzing the relationship between the Seebeck coefficient (*S*) and the carrier concentration (*n*_*H*_) to estimate the density-of-states effective mass (*m*_d_*), as shown in Figure S9(a) (details provided in the Supporting Information). The samples with *x* = 0 to 0.10 closely follow the expected trend based on supplementary equations (S1) and (S2), assuming a constant effective mass. In contrast, the samples with *x* = 0.15 and 0.20 deviate from this trend, most likely due to the influence of the bipolar effect, which alters carrier transport but does not affect the intrinsic band density of states [43]. Although topological effects or magnetic contributions were initially considered as possible influences, no specific features or anomalies related to such phenomena were observed in this study. Therefore, the transport properties can be sufficiently explained using a classical approach based on carrier scattering and phase inhomogeneity.

The temperature-dependent power factor (*PF*) values for the Sr₁_–_*ₓ*Sm*ₓ*Si₂ series are presented in Figure 6(d). A clear enhancement in *PF* is observed with Sm substitution, increasing from 1.9 mW/m.K^2^ at 300 K for undoped SrSi₂ to 2.47 mW/m.K^2^ for *x* = 0.05. The room temperature *PF* of Sr₀.₉₅Sm₀.₀₅Si₂ is higher than those reported in previous studies on SrSi₂ (see Figure S7(b)) [15,19,31,32,44]. Additionally, the Sr₀.₉₅Sm₀.₀₅Si₂ composition exhibits a peak *PF* of 2.9 mW/m.K^2^ at approximately 200 K, marking a more than 50% improvement compared to the peak *PF* of undoped SrSi_2_. In addition, these values are competitive with several established *p*-type silicides [45], half-Heusler, antimonides, and chalcogenides thermoelectric materials at the same temperature range [46]. In our PPMS system measurements, the typical error margin for the Power Factor (*PF*) is estimated to be around 15%, as previously mentioned for electrical resistivity and Seebeck coefficient measurements. These uncertainties affect *PF* calculation and arise from several factors, including instrumental precision, sample geometry measurements, and contact resistance [47,48].

Next, we calculated the Sr_1-*x*_Sm_*x*_Si_2_ samples’ weighted mobility (*μ*_w_) in the 200–350 K temperature range using our experimental data (Figure S8(a)). Snyder et al. [49] proposed an empirical equation to calculate weighted mobility from the Seebeck coefficient and electrical resistivity as follows:

(2) $$\mu_{w}=331\frac{cm^{2}}{\mathrm{Vs}}\frac{m\Omega \mathrm{cm}}{\rho }{\left(\frac{T}{300K}\right)}^{-3\;/\;2}\left[\frac{\exp\left[\frac{\left|S\right|}{k_{B}\mathrm{\setminus e}}-2\right]}{1+\exp\left[-5\left(\frac{\left|S\right|}{k_{B}\mathrm{\setminus e}}-1\right)\right]}\right]+\left[\frac{\frac{3}{\pi^{2}}\frac{\left|S\right|}{k_{B}\mathrm{\setminus e}}}{1+\exp\left[5\left(\frac{\left|S\right|}{k_{B}\mathrm{\setminus e}}-1\right)\right]}\right]$$

The present samples exhibit high-weighted mobilities of ~210 cm^2^/V·s for undoped SrSi₂ and ~285 cm^2^/V·s for Sr_0.95_Sm_0.05_Si_2_ at room temperature, which is notably high compared to other thermoelectric materials, as shown in Figure S8(b). For instance, Bi₂Te₃ alloys typically exhibit *μ*_w_ values of 200–300 cm^2^/V·s, while PbTe demonstrates *μ*_w_ around 100–200 cm^2^/V·s [49,50]. Such a high *μ*_w_ highlights the superior electronic transport properties of the Sm-substituted SrSi₂ system, making it a competitive candidate for thermoelectric applications.

#### 3.2.3. Thermal conductivity

The temperature dependence of the total thermal conductivity *κ*, the electronic thermal conductivity *κ*_e_, and the lattice thermal conductivity *κ*_l_ are shown in Figure 7. The thermal conductivities of Sr_1-*x*_Sm_*x*_Si_2_ samples are 3.2–3.7 W/m.K at 300 K (Figure 7(a)). The current samples showed lower room temperature thermal conductivities than those reported by R. Ghannam et al. [19], and Singh et al. [15], who also utilized the SPS process to fabricate their samples. At low temperatures, *κ* rises with temperature, peaking at 20–40 K due to phonon scattering from point defects. A notable trend in *κ* is the drastic reduction in the height of the low-temperature peak with increasing Sm substitution, indicating enhanced phonon scattering. The undoped SrSi_2_ sample exhibits the lowest *κ* at low temperatures and a diminished phonon peak, attributed to Sr deficiency, confirmed by SEM/EDS analysis. This deficiency enhances phonon scattering by lattice imperfections, similar to trends also shown by Lue et al. [41]. The *κ* of the Sr_1-*x*_Sm_*x*_Si_2_ samples showed an increasing trend from 250 to 350 K, which is attributed to the bipolar diffusion effect. A dip in the *κ* data of Sr_1-*x*_Sm_*x*_Si_2_ samples was observed at 223 K, followed by an increase at higher temperatures. We consider the coexistence of holes and electrons with comparable concentrations. The bipolar diffusion effect was noticed earlier in narrow-band gap systems [51–53]. As Sm substitution in SrSi_2_ introduces an additional electron, it alters the concentration of majority carriers (details in ESI file).

*κ*_e_ was calculated by the Wiedemann–Franz law, *κ*_e_ = *LT/ρ*, where *L* is the Lorenz number. For a degenerate semiconductor, *L* can be estimated from *S* data using the empirical relation [54]:

(3) $$L=1.5+\exp\left[\frac{\left|S\right|}{116}\right]$$

where *S* is in μV/K. The calculated value of *L* increases monotonically with temperature. The *L* value ranges from 1.81 × 10^−8^ to 2.05 × 10^−8^ WΩ/K^2^. The electronic part (*κ*_e_) increased with Sm content at 0.05 and 0.1, then decreased for *x* = 0.15 and 0.2, following the same trend as electrical resistivity.

The lattice thermal conductivity, *κ*_l_, is obtained by subtracting *κ*_e_ from the total thermal conductivity, and the result is presented in Figure 7(b). To clarify the origin of the significant reduction of *κ*_l_ in Sr_1-*x*_Sm_*x*_Si_2_, we fitted the experimental *κ*_l_ data by using the Debye-Callaway approximation [55]:

(4) $$\kappa_{l}=\frac{k_{B}}{2\pi^{2}v_{s}}{\left(\frac{k_{B}T}{\hbar }\right)}^{3}\int_{0}^{\frac{\theta_{D}}{T}}\frac{x^{4}e^{y}}{\tau_{p}^{-1}{\left[e^{y}-1\right]}^{2}}\mathrm{dy}$$

Here, $y=\hbar \omega \;/\;k_{B}T$ is a dimensionless quantity in which *ω*, *ћ*, *k*_B_, *θ*_D_, *υ*_s_, and *τ*_p_ are the phonon frequency, reduced Planck constant, the Boltzmann constant, the Debye temperature, the average phonon velocity, and the phonon scattering relaxation rate, respectively. The relaxation rate is described as [56]:

(5) $$\tau_{p}^{-1}=\frac{v_{s}}{l}+A\omega^{4}+B\omega^{2}\mathrm{Texp}\left(-\frac{\theta_{D}}{3T}\right)$$

The terms on the right-hand side of Equation 5 refer to scattering on grain boundaries with average grain size *l*, point defects, and phonon Umklapp processes, respectively. *A* and *B* are material-dependent constants. The least-squares fit results from Equations 4 and 5 are shown as solid lines in Figure 7(b). As suggested by Pope et al. [57], a term *RT*^*3*^, with R being a fitting parameter, can be added to the least squares fit model as a correction to account for radiation losses at temperatures above 200 K, which inherently arise for steady-state measurement setups, as discussed in [27] essential to the steady state heat flow technique, there is a good agreement between the experimental and theoretical results. There is no single solution for fitting the model; a range of parameter sets can provide a reasonable fit. However, a thorough analysis reveals that the value of R is clearly defined and fluctuates by less than 1% across all the different solutions of the model. This indicates that the *T*^3^ correction is valid and should result in only minor errors. Additionally, incorporating the radiation loss term *RT*^3^, without altering the already refined parameters, improves the fitting near 300 K.

In general, grain-boundary scattering dominates at low temperatures, while the Umklapp process is significant at high temperatures. Point-defect scattering also notably affects the shape and position of the phonon peak in the mid-temperature range. The low-temperature peak in *κ*_l_ is highly sensitive to different mechanisms of phonon scattering in crystalline solids. The parameters obtained from these least-squares fits are tabulated in Table 3. The higher grain-boundary scattering value (*υ*_s_/*l*) in the undoped SrSi_2_ aligns with the smaller grain sizes observed in the EBSD analysis. However, the lack of a systematic trend in the *υ*_s_/*l* values with increasing Sm content suggests that other factors in phonon scattering mechanisms may also play a role in influencing these values [31,56,58]. Moreover, KAM mapping and grain boundary misorientation shown in Figure 5 identify local strain and deformation, correlating it with defect-induced phonon scattering. Higher KAM values often indicate increased defect density, which is beneficial for reducing *κ*_l_ in thermoelectric materials.

**Table 3.** Parameters used to fit the lattice thermal conductivity (*κ*_l_) of Sr_1-*x*_Sm_*x*_Si_2_.

Sr_1−*x*_Sm_*x*_Si_2_	*υ*_*s*_*/l* (10^9^ s^−1^)	*A* (10^42^ s^3^)	*B* (10^−17^ s.K^−1^)
*x* = 0	1.48	11.5	1.26
*x* = 0.05	1.11	7.8	1.62
*x* = 0.10	0.86	8.6	1.36
*x* = 0.15	1.05	9.4	2.1
*x* = 0.20	0.98	10.2	1.9

The point defect scattering parameter *A* (associated with the phonon scattering rate *A*$\left(\tau_{p}^{-1}\propto A\omega^{4}\right)$) exhibits its highest value of 11.5 in the undoped SrSi₂ sample. This is likely attributed to Sr deficiency, as confirmed by EDS analysis, which enhances point defect scattering and leads to suppression of the phonon peak, consistent with previous findings in Sr-deficient alloys reported by Lue et al. [59]. The synthesis of the SrSi_2_ system faces challenges due to Sr deficiency caused by inherent volatility, even with a 5% excess.

Upon Sm substitution, the *A* value initially decreases, reaching 7.8 for *x* = 0.05, indicating a reduction in point defect scattering. This trend indicates that low levels of Sm incorporation contribute to stabilizing the lattice by reducing Sr volatility and minimizing vacancy formation. This is further supported by the improved compositional homogeneity shown in Table 1, as well as by previous studies that demonstrate enhanced structural stability and decreased vacancy formation resulting from cationic doping [60–62].

However, with further Sm substitution, the *A* parameter shows a gradual increase, rising to 10.2 at *x* = 0.20 (Table 3). This behavior may be attributed to the onset of additional phonon scattering arising from mass fluctuation and local lattice strain induced by higher Sm content. These results indicate a delicate balance: while initial Sm substitution compensates for intrinsic structural deficiencies, excessive substitution introduces new scattering sources, influencing the overall phonon transport.

The efficiency of point defect scattering arises from the combined effects of mass fluctuations and volume variances of atoms. In general, this efficiency can be quantified using a disorder parameter *Г*, expressed as *Г* = *Г*_*m*_ + *Г*_*s*_, where the subscripts *m* and *s* represent mass and strain field, respectively. The disorder parameter *Γ* for Sr_1-*x*_Sm_*x*_Si_2_ samples was estimated following the method described in Ref [63], and the data for relative atomic mass and covalent radius were obtained from Ref [64]. Figure S7(a) shows that as the Sm content increases, the strain field fluctuations (*Γ*_s_) have a limited effect because of the relatively smaller covalent radius differences between Sr (*R*_Sr_ = 1.95 Å) and Sm (*R*_Sm_ = 1.98 Å). On the other hand, the mass fluctuations (*Γ*_m_) have a more significant effect on lowering *κ*_l_ due to the more considerable mass difference between them (*M*_Sr_ = 87.52 and *M*_Sm_ = 150.36). This observation highlights the importance of point-defect scattering in the lattice thermal conductivity of substituted SrSi_2_ alloys. However, we did not observe any systematic changes in the Umklapp coefficient *B* from our fits. We conclude that the variation in lattice thermal conductivity at low temperatures in these alkaline-earth-metal silicides is likely due to modifications in the point-defect scattering mechanism.

The quality factor *μ*_W_/*κ*_l_, which is the ratio of weighted mobility and lattice thermal conductivity, serves as a reliable indicator of thermoelectric performance [65]. High-weighted mobility and low lattice thermal conductivity are essential to achieving outstanding thermoelectric performance. The calculated values of *μ*_W_/*κ*_l_ are shown in Figure 7(d). At room temperature, the *μ*_W_/*κ*_l_ of Sr_0.95_Sm_0.05_Si_2_ exhibits a substantial increase of ~ 60% compared with that of undoped SrSi_2_. This increase illustrates the strategic tailoring of scattering centers to reduce lattice thermal conductivity and enhance carrier mobilities. The present *μ*_W_/*κ*_l_ ratio for the Sr_0.95_Sm_0.05_Si_2_ sample(~135 at 300 K) is comparable to many well-established high-performance thermoelectric materials near room temperature, such as (Bi, Sb)_2_(Te, Se)_3_, Mg_3_(Sb, Bi)_2_, MgAgSb, AgSbTe_2_, Ag_2_Se, CsBi_4_Te_6_, and SnSe-based materials, which shows values around 100–150 [49,65].

#### 3.2.4. Figure of merit

**Figure 8.**
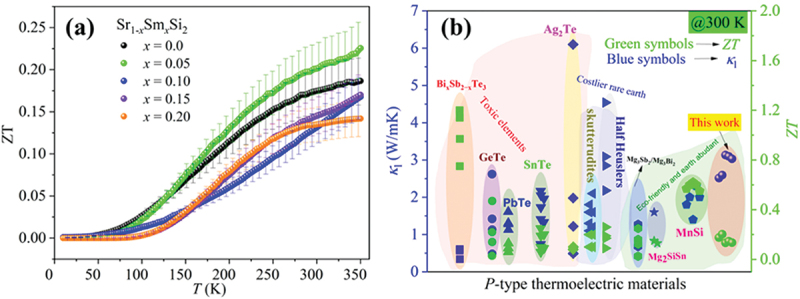
(a) Temperature-dependent Figure of merit *ZT* for the Sr_1-*x*_Sm_*x*_Si_2_ sample. (b) Compare room temperature *κ*_*l*_ and *ZT* values for representative *p*-type TE materials [66]. The uncertainty in the calculated thermoelectric figure of merit (*ZT*) is estimated to be ± 20%, assuming ± 5% errors in *κ*, *S*, and resistivity (*ρ*), with negligible contribution from temperature measurement errors.

Figure 8(a) presents the temperature dependence of the *ZT* value for Sr₁_–_*ₓ*Sm*ₓ*Si₂. The *ZT*_max_ values of about ~ 0.17 and ~ 0.23 for undoped SrSi₂ and Sr₀.₉₅Sm₀.₀₅Si₂, respectively, were obtained at 350 K. The *ZT* value at 300 K for undoped SrSi₂ exceeds those reported in SrSi_2_, as shown in Figure S7(b) [16,31,42]. The approximately 35% improvement in *ZT* compared to undoped SrSi₂ is due to an optimized balance between electrical resistivity and the Seebeck coefficient. This balance has been achieved by precisely tuning the Fermi level and suppressing the bipolar effect, even though there is a modest decrease in thermal conductivity. In comparison to other *p*-type thermoelectric materials, Sr₀.₉₅Sm₀.₀₅Si₂ exhibits competitive performance. For example, *p*-type ZnSb and CoSb_3_-skutterudites typically display *ZT* values of approximately 0.1 to 0.15 and around 0.2 at 300 K, respectively. In contrast, Bi₂Te₃, considered a benchmark thermoelectric material, achieves a *ZT* of about 1.0 under similar conditions. Although the *ZT* of Sr₀.₉₅Sm₀.₀₅Si₂ is lower than that of Bi₂Te₃, it presents the advantages of utilizing earth-abundant, non-toxic constituents and scalable synthesis methods. Figure 8(b) compares room temperature *ZT* and *κ*_l_ values for representative *p*-type TE materials with the present work [66]. These findings position Sm-substituted SrSi₂ as a promising candidate for mid-range thermoelectric applications, blending environmental sustainability with cost-efficiency.

## 4. Conclusions

This study systematically investigates the effect of Sm substitution on the thermoelectric properties of SrSi₂. The room-temperature electrical resistivity and thermal conductivity show a substantial reduction upon the substitution of Sm (*x* < 0.10) onto the Sr sites of SrSi_2_. However, the Seebeck coefficients decreased due to substitution (*x* < 0.10). The reduced electrical resistivity and moderate Seebeck coefficient result in a considerably improved power factor. The *PF* of 2.47 mW/m.K^2^ observed in Sr_0.95_Sm_0.05_Si_₂_ at 300 K is competitive with several established *p*-type thermoelectric materials at room temperature. Moreover, the maximum *PF* of 2.9 mW/m.K^2^ at around 200 K for Sr_0.95_Sm_0.05_Si_2_ corresponds to more than 50% enhancement than the peak value for SrSi_2._ The high *PF* combined with lower thermal conductivity enhances the Figure of merit (*ZT*) in Sr₀.₉₅Sm₀.₀₅Si₂ (*ZT* ~0.23 at 350 K), representing a ~ 35% improvement over pristine SrSi₂. Despite these advancements, further reduction in thermal conductivity remains a challenge. Nanostructuring and multiscale defect engineering could further optimize phonon scattering while preserving favorable electronic properties. These findings establish Sm-substituted SrSi₂ as a promising and scalable thermoelectric material derived from earth-abundant elements, contributing to the ongoing search for sustainable energy conversion technologies.

## Supplementary Material

Supplemental Material

## Data Availability

Data will be made available on request.
