# Supplementary material for: Optimization of thermoelectric performance in Sm-substituted SrSi₂ via carrier transport and lattice engineering
Source: Sci Technol Adv Mater. 2025 Aug 27;26(1):2551486. doi: 10.1080/14686996.2025.2551486 (PMC12447468; doi:10.1080/14686996.2025.2551486)
Supplement: Supplemental Material [file TSTA_A_2551486_SM0155.docx]

**Supporting Information**

**Optimization of thermoelectric performance in Sm-substituted SrSi₂ via carrier transport and lattice engineering**

Vikrant Trivedi^1^, Naohito Tsujii^1*^, Takao Mori^1,2*^

1 Research Center for Materials Nanoarchitectonics (MANA),

National Institute for Materials Science (NIMS), Namiki 1-1, Tsukuba, 305-0044, Japan.

2 Graduate School of Pure and Applied Sciences, University of Tsukuba,

Tennodai 1-1-1, Tsukuba 305-8671, Japan.

*E-mail of the corresponding author: [TSUJII.Naohito@nims.go.jp](mailto:TSUJII.Naohito@nims.go.jp), [MORI.Takao@nims.go.jp](mailto:MORI.Takao@nims.go.jp)

**
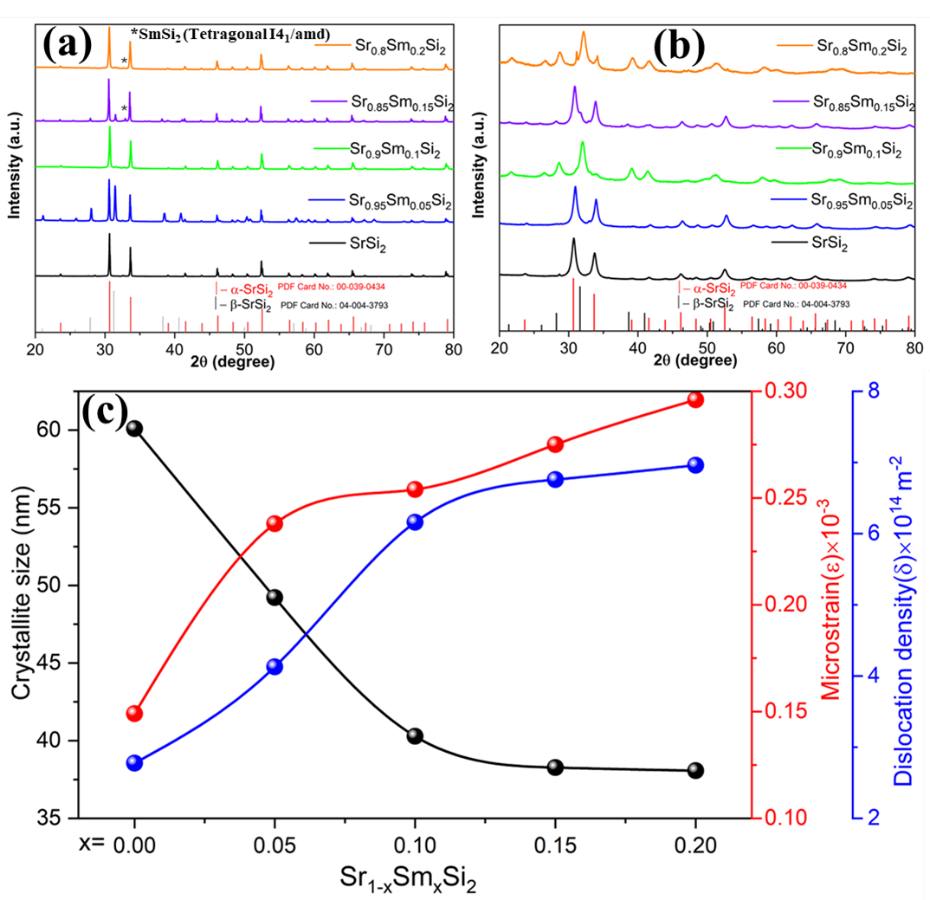
**

**Figure S1:** X-ray diffraction pattern of (a) arc melted and (b) ball milled Sr_1-x_Sm_x_Si_2_ samples, respectively, with the indexation of the *α*-SrSi_2_ and *β*-SrSi_2_ phases, Impurity peaks at *x* = 0.15 and 0.2, marked by asterisks. (c) All the samples' dislocation density, micro-strain, and crystallite size were calculated using a modified Williamson-Hall (W-H) method from the XRD data.

**
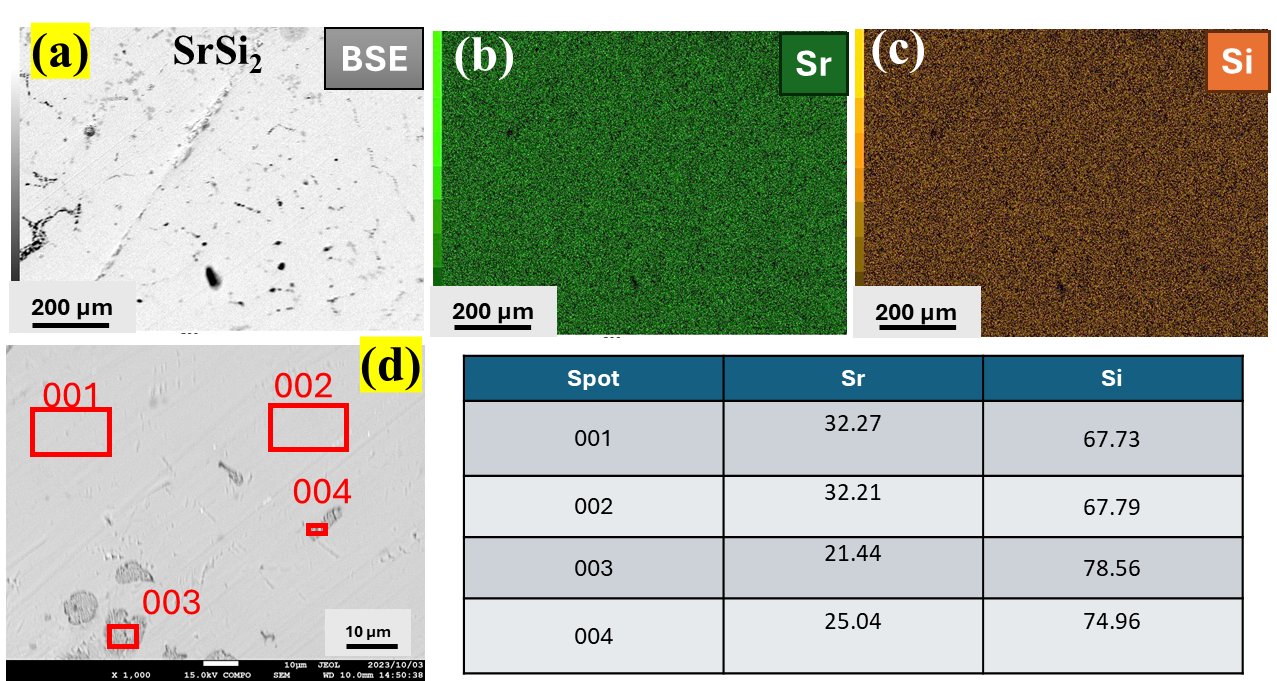
**

**Figure S2:** (a, d) BSE images of arc melted SrSi_2_, and (b, c) the respective EDS elemental mapping of (a) and composition table with respective spots in (d).


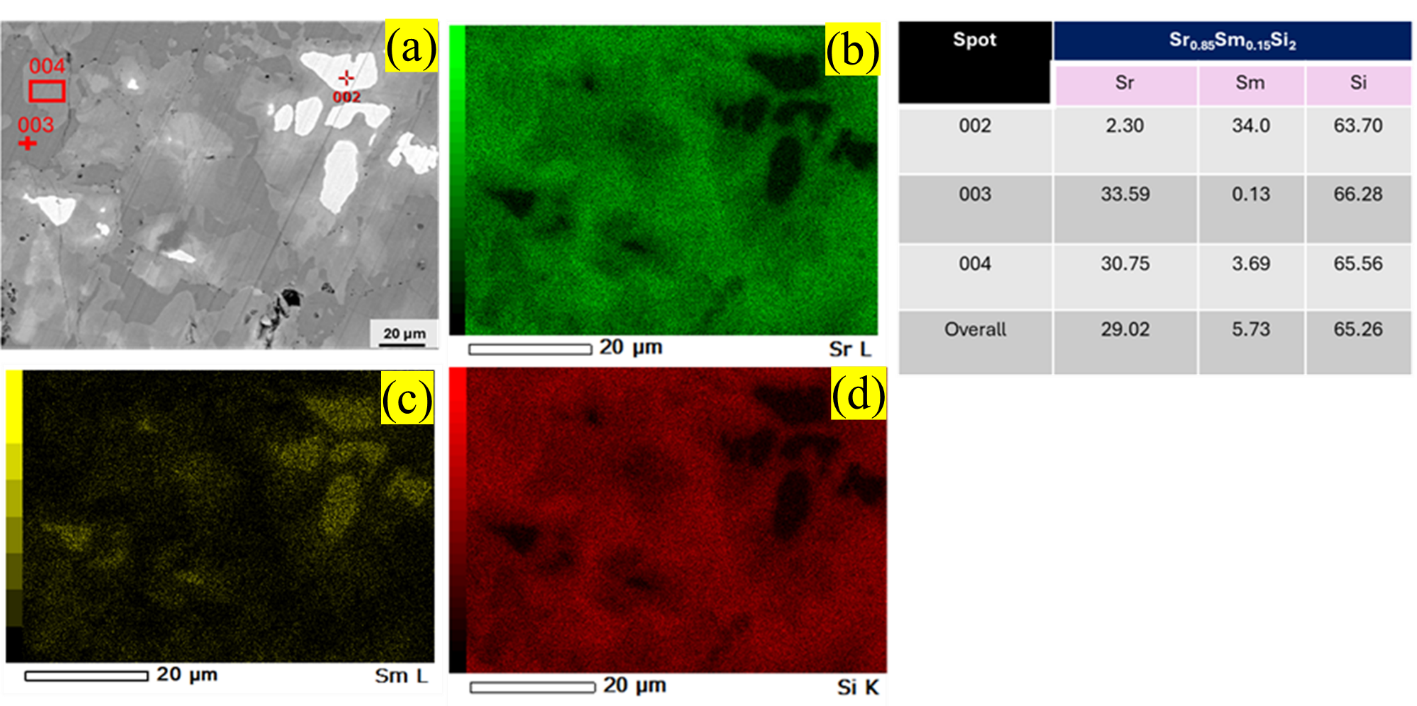


**Figure S3:** (a) BSE images of arc melted Sr_0.85_Sm_0.15_Si_2_, and (b, c, and d) the respective EDS elemental mapping and composition table with respective spots in (a).


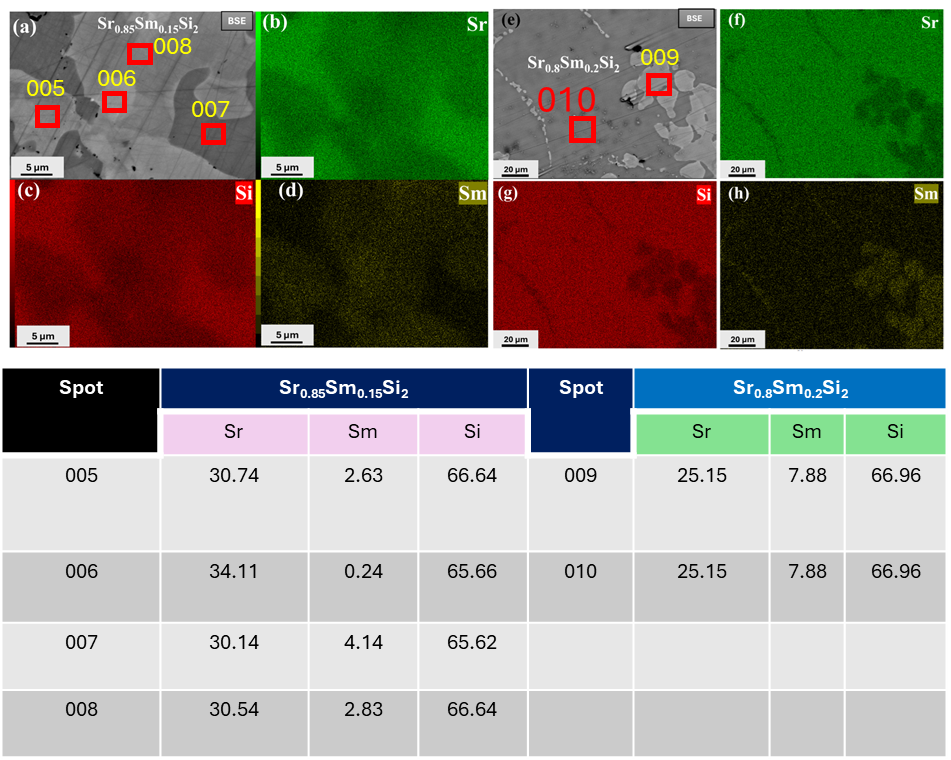
**Figure S4**: BSE images of SPSed Sr_0.85_Sm_0.15_Si_2_ (a) and Sr_0.8_Sm_0.2_Si_2_ (e), and the respective EDS elemental mapping and respective compositions from selected area spots (b-d) for Sr_0.85_Sm_0.15_Si_2_ and (f-h) for Sr_0.8_Sm_0.2_Si_2_.

**M1. Modified Williamson-Hall (W-H) method for crystallite size and dislocation density**

The XRD data was further analyzed using a modified Williamson-Hall (W-H) method to estimate the crystallite size, dislocation density, and lattice strain. The analysis employed Convolutional Multiple Whole Profile (CMWP) fitting algorithms, with a Si-standard utilized to calculate the instrumental broadening. Assuming isotropic strain in the material, the W-H equation for the total peak broadening is as follows [1]:

$\beta_{hkl}cos\theta_{hkl}=\frac{k\lambda}{D_{v}}+4\epsilon sin\theta_{hkl}$ and $\delta=\frac{1}{D_{v}}$ Eq (S1)

In the given equation, *k* represents the shape factor (usually taken as 0.9), *β*_hkl_ is the instrumental correction for peak broadening, *θ*_hkl_ is the Bragg angle corresponding to the (hkl) plane, and *D*_V_ is the volume-weighted crystallite size. The dislocation density (*δ*) was calculated as the inverse of the volume-weighted crystallite size. According to the Williamson-Hall model, lattice strain and crystallite size were determined from the slope and intercept of the linear plot between *β*_hkl_cos *θ*_hkl_ and 4 sin*θ*_hkl_.


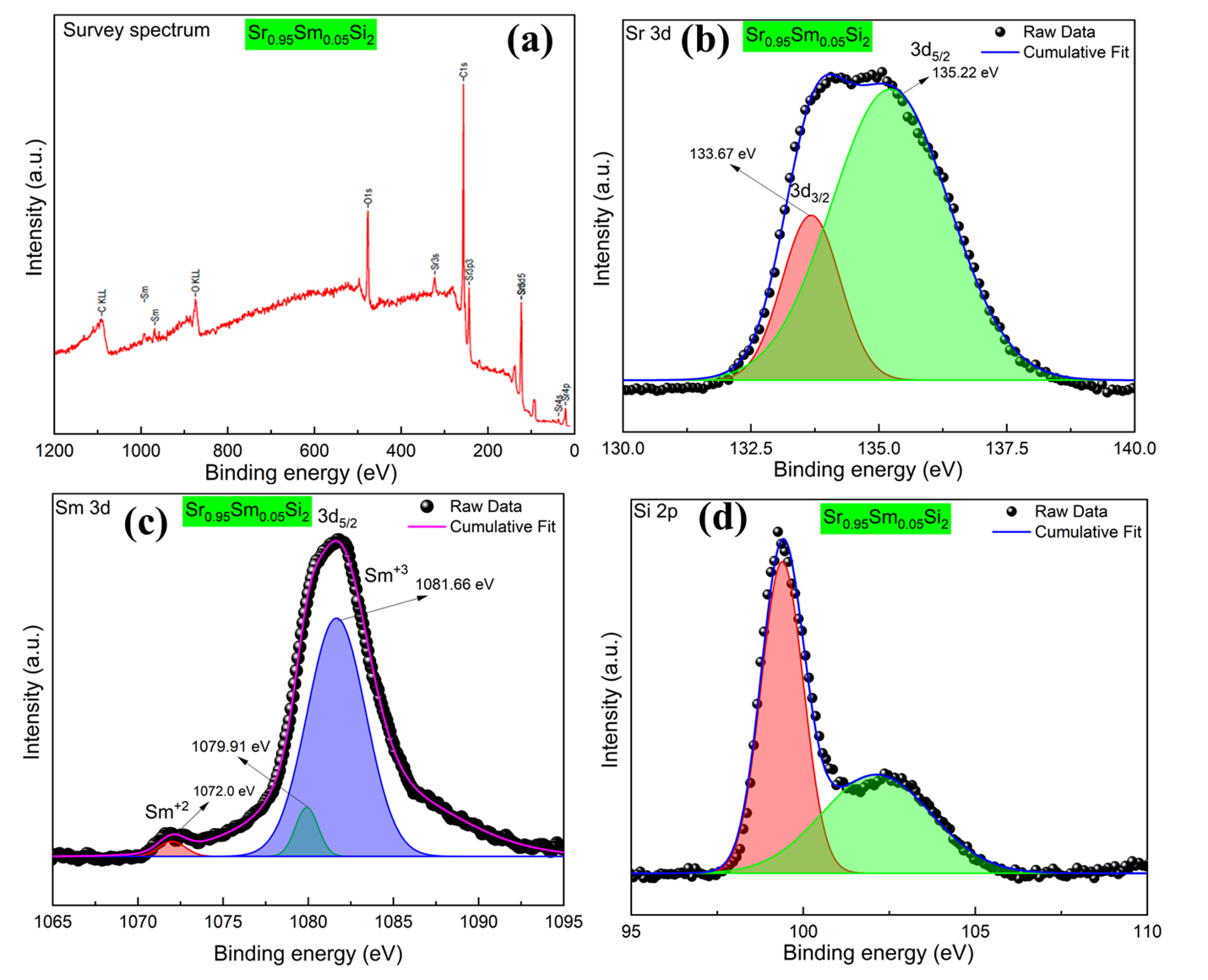


**Figure S5:** High-resolution core level XPS spectra : (a) XPS survey spectrum of SPSed Sr_0.95_Sm_0.05_Si_2_ sample, (b) Sr3d spectrum; (c) Sm 3d; (d) Si2p deconvolution demonstrating their chemical states and binding energies.


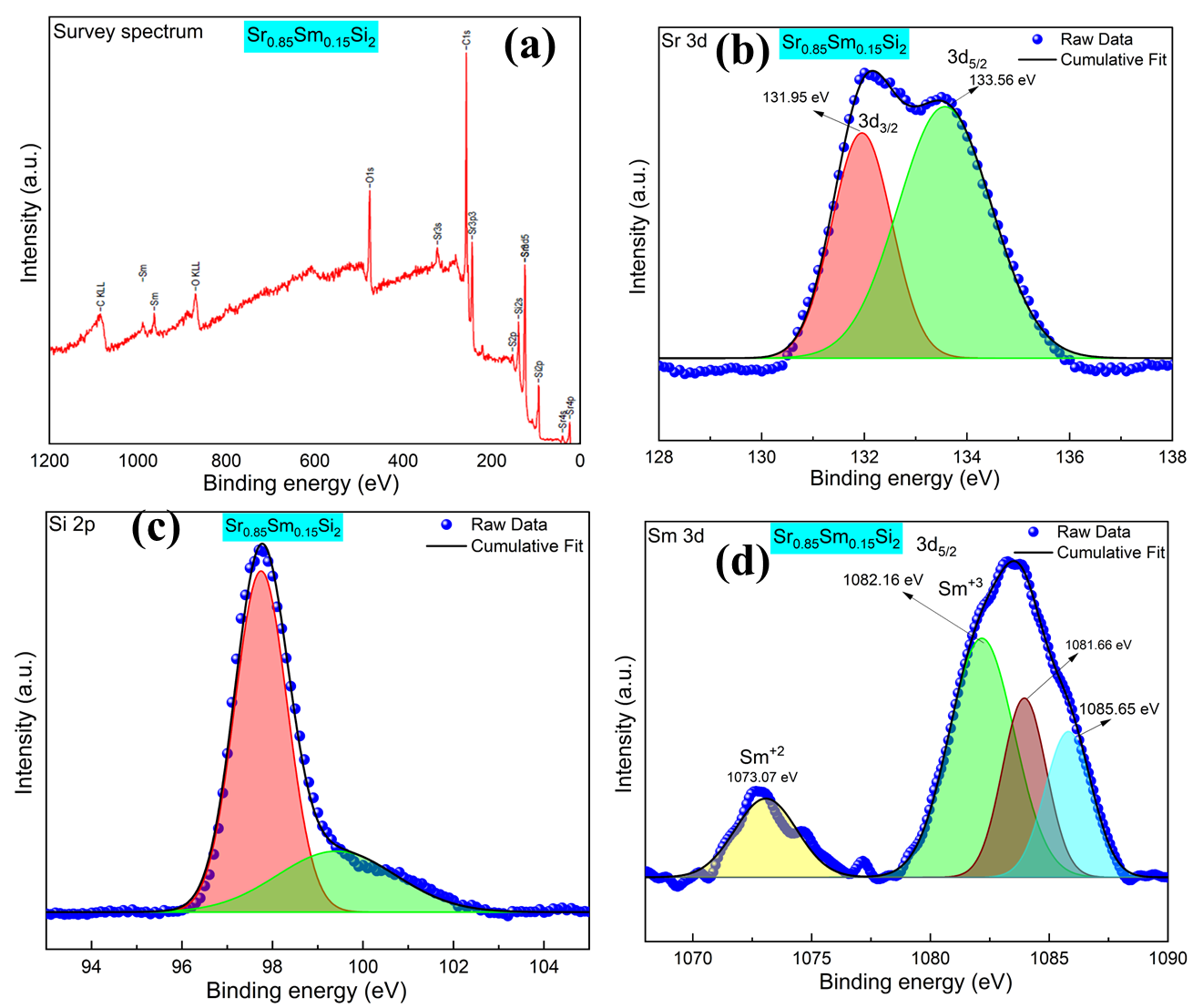


**Figure S6:** High-resolution core level XPS spectra : (a) XPS survey spectrum of SPSed Sr_0.85_Sm_0.15_Si_2_ sample, (b) Sr3d spectrum; (c) Sm3d; (d) Si2p deconvolution demonstrating their chemical states and binding energies.


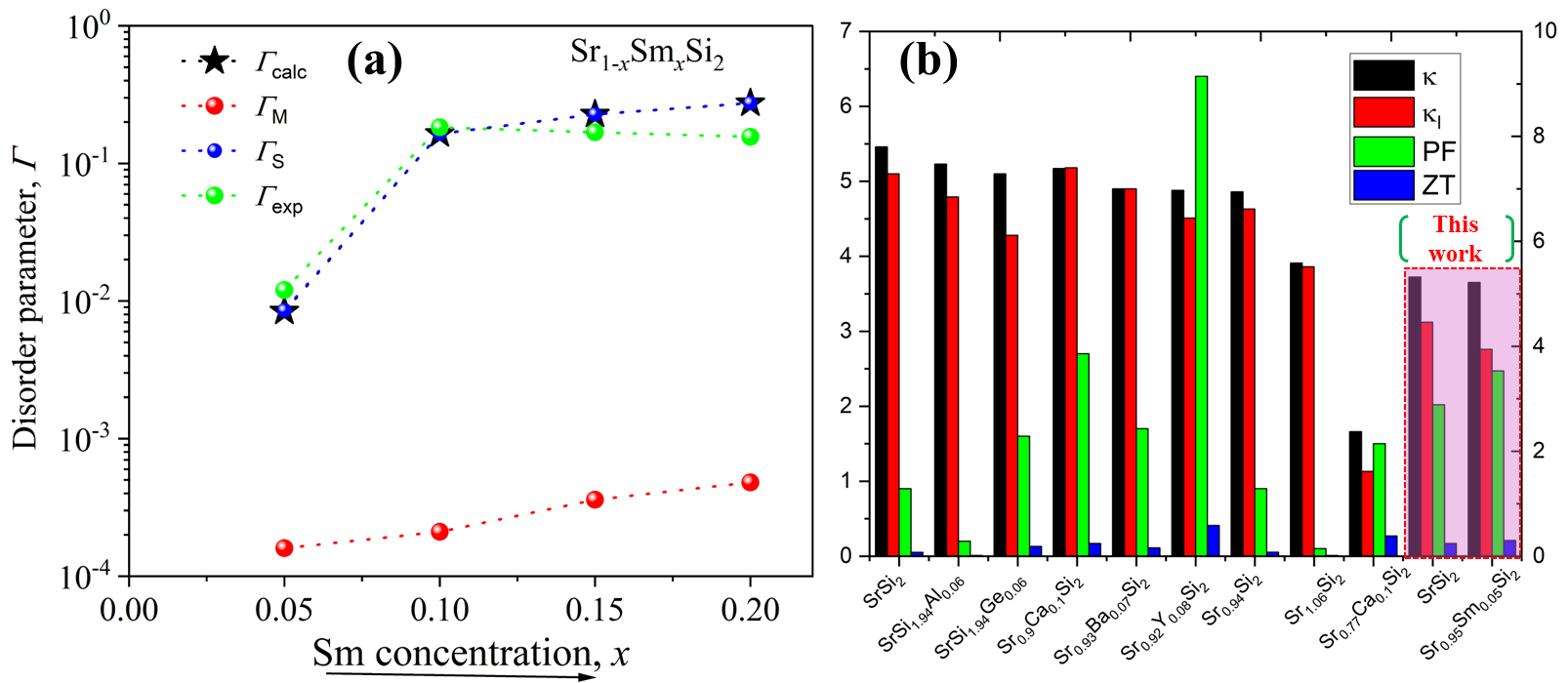


**Figure S7:** (a) concentration-dependent disorder parameter (*Г*) of Sr_1-x_Sm_x_Si_2_ derived experimentally (*Г*_exp_) and calculated (Г_calc_), which includes the mass fluctuations(*Г*_m_) and strain contribution (*Г*_s_); (b) the *ZT* for the SrSi_2_ and Sr_0.95_Sm_0.05_Si_2_ samples compared with the previously reported typical SrSi_2_ materials.

**
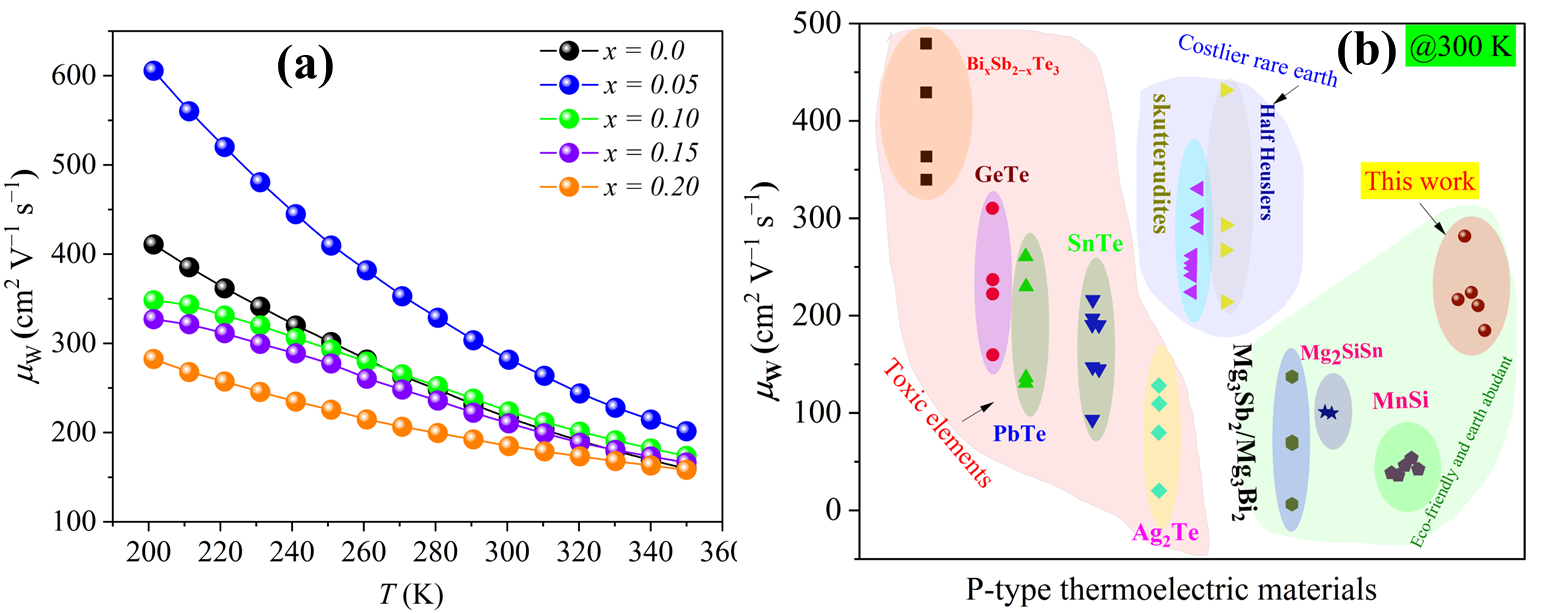
**

**Figure S8:** (a) Temperature-dependent weighted mobility, (b) comparison graph of weighted mobility of some *p*-type thermoelectric materials with the current work [4].


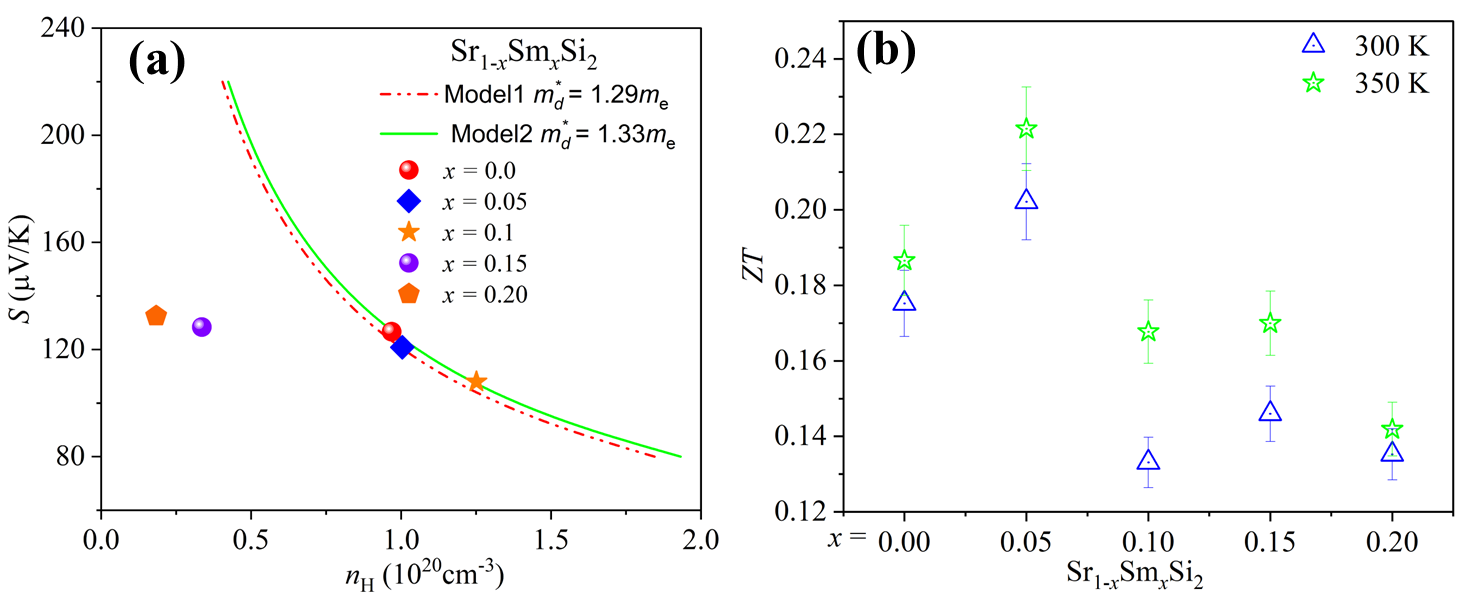


**Figure S9:** Pisarenko plot showing the absolute value of *S* with *n_H_*. The solid lines and the dotted lines represent model 1 and model 2, respectively. (b) *ZT* at 300 K and 350 K for the present samples, respectively.

**M2: Determine the Density-of-States Effective Mass with Carrier Concentration-Dependent Seebeck Coefficient (Pisarenko relation**):

To better explain the impact of Sm substitution, we utilized the Pisarenko relation between *S* and *n*_H_ by estimating *m*_d_*. Based on a single parabolic band (SPB) model and assuming energy-independent carrier scattering for degenerate semiconductors, *S* can be described by Eq. (S2) [2]:

$S= {8\pi^{2}k_{B}^{2}Tm_{d}^{*}}/{3eh^{2}\left( \pi/{3n} \right)^{\frac{2}{3}}}$ Eq (S2)

Where *k*_B_, *e*, *h*, and *n* are the Boltzmann constant, the electronic charge, the Planck constant, and the carrier concentration, respectively. We also used a simplified equation for calculating *m*_d_* given as:

$\log_{10} \left( \frac{m_{d}^{*}T}{300} \right)= \frac{2}{3}\log_{10} (n_{H})-\frac{2}{3}[20.3-\left( 0.00508\times\left| S \right| \right)+(1.58\times{0.967}^{\left| S \right|})$ Eq (S3), Where *n*_H_ represents the measured carrier concentration. To avoid confusion about calculating *m*_d_* using eq S2 and S3, we call eq S2 and S3 models 1 and 2, respectively.

**M3: Bipolar effect on thermoelectric materials**

According to the mass action law in semiconductors, under thermal equilibrium, the product of hole and electron concentration is the square of the intrinsic carrier concentration independent of the doping level:

$n_{e}n_{h}=n_{i}^{2}=N_{C}N_{V}=exp\left( \frac{-E_{g}}{k_{B}T} \right)$ Eq (S4)

*N*_C_ and *N*_V_ are the electron and hole-effective density of states, respectively. Therefore, the concentration of minority carriers depends on the band gap, *E*_g_, temperature *T*, and the concentration of majority carriers. This suggests that at certain temperatures, the bipolar effect can be suppressed by either increasing the band gap or increasing the majority carrier concentration.

In the case of thermal conductivity, we consider the coexistence of hole and electron with equal carrier concentrations of *n*_h_ and *n*_e._ *κ*_e_ can be the sum of the Wiedemann–Frantz law and bipolar diffusion effect. This bipolar contribution can be signified as [3]:

$\kappa_{bi}= \frac{b}{\left( b+1 \right)^{2}}\left[ \frac{E_{g}}{k_{B}T}+4 \right]^{2}\left( \frac{k_{B}}{e} \right)^{2}\sigma_{i}T$ Eq S (5)

Where b is the ratio of electron mobility to hole mobility, and σ_i_ is the intrinsic regime electrical conductivity. The equation can be simplified as $\kappa_{bi}=F_{bi}T^{p} exp\left( \frac{-E_{g}}{{2k}_{B}T} \right)$ , where *F*_bi_ and *p* are variable parameters, varying with doping type.

**Table S1**: Lattice parameter, crystallite size, and relative density of the SPS processed samples.

| ***x*** | **Lattice parameter (Å)** | **Crystallite size (nm)** | **Relative density (%)** | **Microstrain**  **(*ε* ×10^-3^)** |  |
| --- | --- | --- | --- | --- | --- |
| **0.00** | 6.535(2) | 60.0 | 97.3 | 0.149 |  |
| **0.05** | 6.536(3) | 49.2 | 98.2 | 0.238 |  |
| **0.10** | 6.537(1) | 40.2 | 96.8 | 0.254 |  |
| **0.15** | 6.5372(5) | 38.2 | 97.5 | 0.275 |  |
| **0.20** | 6.5377(6) | 38.0 | 98.3 | 0.296 |  |

**References:**

[1] Hassanzadeh-Tabrizi SA. Precise calculation of crystallite size of nanomaterials: A review. J Alloys Compd.2023;**968**:171914.https://doi.org/10.1016/j.jallcom.2023.171914

[2] Lee KH, Kim S, Lim J, et al. Approach to Determine the Density‐of‐States Effective Mass withcharge-Dependent Seebeck Coefficient. Adv Funct Mater . 2022;**32**(33):2203852.https://doi.org/10.1002/adfm.202203852.

[3] Yelgel ÖC, Srivastava GP.Thermoelectric properties of n-type Bi_2_(Te_0.85_Se_0.15_)_3_ single crystals substituted with CuBr and SbI_3_.Phys Rev B.2012;**85**(12):125207. https://doi.org/10.1103/PhysRevB.85.125207.

[4] Freer R, Ekren D, Ghosh T, et al. Key properties of inorganic thermoelectric materials—tables (version 1). Journal of Physics: Energy. 2022;**4**(2):022002.https://doi.org/10.1088/2515-7655/ac49dc.
